# Effects of glucose-insulin infusion during major oral and maxillofacial surgery on postoperative complications and outcomes

**DOI:** 10.1186/s40981-018-0148-3

**Published:** 2018-01-22

**Authors:** Akina Tohya, Atsushi Kohjitani, Sachi Ohno, Kaoru Yamashita, Yozo Manabe, Mitsutaka Sugimura

**Affiliations:** Department of Dental Anesthesiology, Field of Oral and Maxillofacial Rehabilitation, Graduate School of Medical and Dental Sciences, Kagoshima University, 8-35-1 Sakuragaoka, Kagoshima, 890-8544 Japan

**Keywords:** Surgical diabetes, Inflammation, Hypoalbuminemia, Postoperative complication

## Abstract

**Background:**

Secretion of hormones, which antagonize the action of insulin, is facilitated in response to surgery, and acute resistance to the action of insulin develops. Our aim is to elucidate the effects of intraoperative glycemic control by glucose-insulin (GI) infusion on postoperative complications and outcomes in major oral and maxillofacial surgery.

**Findings:**

Thirty patients aged ≥ 60 years undergoing a radical operation of oral malignant tumors with tissue reconstruction (≥ 8 h) were analyzed. In the GI group, regular insulin was continuously applied with glucose-added acetate Ringer’s solution (5–10 g glucose per 500 mL). Blood glucose was adjusted within the target concentration of 80–120 mg/dL. In the control group, combination of acetate Ringer’s solution containing 1% (*W*/*V*) glucose and lactate Ringer’s solution, which contains no glucose, was employed. Perioperative clinical parameters, incidence of hypoalbuminemia, and postoperative complications, i.e., surgical site infection, necrosis of a reconstructed flap, bacteremia, hypotension, or pneumonia, were compared. Both serum total protein and albumin concentrations (postoperative day 1 [Day1]) were higher in the GI group. The mean infusion rate of glucose during surgery (mg/kg/h) was independently associated with the decrease in both serum total protein and albumin concentrations from the control to Day1. No difference was found between the groups in the incidence of postoperative complications and the days required until discharge, except less incidence of hypoalbuminemia in the GI group.

**Conclusions:**

Application of additional glucose during major oral and maxillofacial surgery preserved serum albumin concentration. However, it did not lead to less postoperative complications and less days until discharge.

## Introduction

The neuroendocrine system facilitates the secretion of adrenaline, growth hormone, cortisol, and adrenocorticotropic hormone (ACTH) in response to surgical stimulation, all of which antagonize the action of insulin. These counter-regulatory hormones augment gluconeogenesis in the liver, inhibit glucose uptake into cells, and interfere with insulin secretion from pancreatic β-cells, which leads to a state called “surgical diabetes” [1]. Furthermore, acute resistance to the action of insulin also develops during surgery [2, 3], especially in skeletal and cardiac muscle, adipose tissue, and the liver, where glucose uptake is regulated by glucose transporters [4].

Hyperglycemia triggers and amplifies a cascade of inflammatory responses. Hyperglycemia facilitates production of superoxide anions through activation of nicotinamide adenine dinucleotide phosphate (NADPH) oxidase [5, 6]. Superoxide anions in turn trigger production of inflammatory cytokines, such as interleukin (IL)-1β, IL-6, or tumor necrosis factor (TNF)-α [7], which augment both inflammatory responses and insulin resistance. Meanwhile, insulin exhibits anti-inflammatory actions by reducing production of inflammatory cytokines and reactive oxygen species [8].

Recently, control of blood glucose during the perioperative period has been emphasized because high blood glucose delays normal healing of surgical wounds and increases the risk of infection. Evidences revealed that tight glycemic control reduced morbidity and mortality of critically ill patients [9] and decreased infection rate and improved survival of coronary bypass graft surgery [10, 11]. Furthermore, the anti-inflammatory effect of intensive insulin therapy during cardiac surgery has been demonstrated [12].

In the current study, we investigated the effects of intraoperative glycemic control by glucose-insulin (GI) infusion on postoperative complications and outcomes in elderly patients undergoing major oral and maxillofacial surgeries, which require 8 h or longer.

## Methods

Thirty-nine patients diagnosed with oral malignant tumors who were aged ≥ 60 years and scheduled for radical operation with tissue reconstruction (scheduled time required ≥ 8 h) from February 2013 to May 2016 were enrolled following approval of the Clinical Ethical Committee of Kagoshima University Hospital. The study was further registered with the UMIN Clinical Trials Registry (UMIN-CTR), UMIN000015522. Written informed consent was obtained from all patients. Patients were category I (normal healthy patients, no organic, physiological, or psychiatric disturbances) or II (patients with mild systemic disease, no functional limitations) of the American Society of Anesthesiologists physical status classification [13]. Patients were randomly allocated to the GI and control groups using computer software that generated random whole numbers, where odd numbers were assigned to the control group and even numbers to the GI group. Patients who had diabetes mellitus, who were not able to continue GI infusion due to hypoglycemia, or whose actual operation time was less than 8 h, were excluded from the analysis.

Anesthesia was induced by propofol (2 mg/kg) or thiopental sodium (5 mg/kg) and inhalation of sevoflurane with 66% nitrous oxide in oxygen. Tracheal intubation was then facilitated by intravenous vecuronium (0.1 mg/kg) or rocuronium (1 mg/kg). In some cases, tracheotomy was performed under local anesthesia and then general anesthesia was induced using the same anesthetics that were described above. Anesthesia was maintained by sevoflurane and nitrous oxide in oxygen combined with an opioid analgesic, remifentanil (0.05–0.3 μg/kg/min). Patients were ventilated to maintain an end-tidal concentration of carbon dioxide at 35–40 mmHg. Routine patient monitoring included non-invasive blood pressure, invasive blood pressure through the radial artery, electrocardiogram, pulse oximetry, and an inspired/expired anesthetic gas and carbon dioxide, which were included in a patient monitor SOLAR 8000 (GE Marquette Medical Systems). Adequate analgesia was achieved by remifentanil, keeping the blood pressure of around 100 mmHg and heart rate of 80 beats/min; hypertension > 140 mmHg and tachycardia > 100 beats/min were avoided. Blood transfusion was performed when the hemoglobin concentration was < 8.0 g/dL or unstable systolic blood pressure was < 80 mmHg.

In the control group, combination of acetate Ringer’s solution which contains 1% (*W*/*V*) glucose and lactate Ringer’s solution, which contains no glucose, was infused. Regular insulin was subcutaneously applied each time when a blood glucose concentration of ≥ 180 mg/dL occurred. In the GI group, 1–4 U/h of regular insulin was continuously applied with infusion of glucose-containing acetate Ringer’s solution (5–10 g glucose per 500 mL) during surgery. In the GI group, blood glucose was adjusted within the target concentration of 80–120 mg/dL. Measurement of blood glucose concentrations was performed every 30 min during the operation. When the infusion rate of insulin was changed, blood glucose concentrations were measured every 15 min to avoid hypoglycemia. Briefly, in most cases, insulin was infused at 1–3 U/h for maintaining target glucose concentrations when acetate Ringer’s solution, in which 10 g glucose was contained, was infused at a constant speed (e.g.*,* 250 mL/h for 50 kg body weight) throughout surgery. A second venous route was used to provide additional fluid (containing no glucose) when volume load was required. In both groups, low-dose dopamine (3 μg/kg/min) was simultaneously administered to maintain renal blood flow and to obtain constant urine output. Arterial blood samples were analyzed every 2–3 h to prevent hypokalemia. Potassium chloride was supplemented (approximately 20 mEq/h) to achieve a target plasma concentration of 4–4.5 mEq/L when hypokalemia (< 3.5 mEq/L) was observed.

Pre- and postoperative C-reactive protein (CRP), body temperature measured in the armpit, total protein (TP) and serum albumin concentration, days until discharge, and incidence of postoperative hypoalbuminemia and complications were compared. Postoperative complications, i.e., surgical site infection (SSI) including methicillin-resistant *Staphylococcus aureus* (MRSA), necrosis of a reconstructed flap, bacteremia, hypotension, or pneumonia, indicated those which required additional therapeutic interventions. The highest body temperature was used when multiple measurements were performed within a day. Values of blood examinations at postoperative day 4 (Day4) or Day5 are represented as Day4/5, using the higher values of CRP and body temperature and the lower values of TP and serum albumin, when data at both days were available.

### Statistical analysis

Data are expressed as means with standard deviation (SD). One-way analysis of variance was used to test significance between the two groups in the baseline numerical items, in the CRP concentration and body temperature for each time point, and days required until discharge. The chi-square test was used to test differences between the groups for baseline categorical items (sex, history of hypertension, dyslipidemia, and smoking habit), incidence of each postoperative complication, and number of patients who had postoperative complications. Correlations between the mean infusion rate of glucose applied during surgery (*X*) and TP and serum albumin concentrations (*Y*) were analyzed by Pearson’s correlation coefficient, and relations were fitted by simple regression. Multiple linear regression analysis was performed to estimate the dependence of age, body mass index (BMI), mean infusion rate of glucose (mg/kg/h) and insulin (mU/kg/h) during surgery, operation time, total fluid volume, intraoperative blood loss, and intraoperative blood transfusion on the reduction of TP and albumin concentrations from the control to Day1 values. *P* < 0.05 was considered statistically significant.

## Results

The total number of patients enrolled in the study was 39. Nine patients were excluded from the data analysis due to short operation time less than 8 h (3 patients in the GI group), hypoglycemia during GI infusion (1 patient in the GI group), and diabetes mellitus (3 patients in the control group and 2 patients in the GI group). Thus, the total number of patients included in the data analysis was 30; the control group comprised 20 patients, and the GI group comprised 10 patients. Baseline patient characteristics are shown in Table 1. There were no differences in the clinical parameters between the two groups, except for the mean infusion rate of glucose and insulin during surgery.

**Table 1:** Baseline characteristics of the patients

	Control group (*N* = 20)	GI group (*N* = 10)	*P* value
Age (years)	72.7 ± 8.4	77.0 ± 8.2	0.2010
Sex (M/F)	12/8	7/3	0.5891
Weight (kg)	52.0 ± 8.0	50.1 ± 12.3	0.6171
BMI (kg/m^2^)	21.4 ± 2.5	20.3 ± 3.6	0.3147
SBP (mmHg)	132.6 ± 21.9	130.2 ± 12.3	0.7468
DBP (mmHg)	70.0 ± 9.7	71.7 ± 6.9	0.6259
HR (/min)	70.3 ± 9.4	71.0 ± 6.1	0.8453
Hypertension	13 (65.0%)	4 (40.0%)	0.1928
Dyslipidemia	6 (30.0%)	1 (10.0%)	0.1976
Current or past smoking	9 (45.0%)	2 (20.0%)	0.1685
Fasting plasma glucose (g/dL)	90.2 ± 10.4	90.8 ± 8.3	0.8894
Triglyceride (mg/dL)	111.5 ± 56.7	83.1 ± 30.8	0.1522
Total cholesterol (mg/dL)	188.4 ± 32.8	185.5 ± 31.6	0.8194
BUN (mg/dL)	14.0 ± 4.9	16.9 ± 4.0	0.1266
Serum creatinine (mg/dL)	0.72 ± 0.2	0.70 ± 0.1	0.8518
eGFR (mL/min/1.73m^2^)	79.7 ± 15.5	78.0 ± 19.1	0.8156
Duration of operation (min)	744.3 ± 168.3	709.8 ± 167.6	0.6004
Duration of anesthesia	903.3 ± 158.4	845.0 ± 178.2	0.3695
Total infusion (mL)	4307 ± 970	3759 ± 1284	0.2015
Blood transfusion (mL)	192.0 ± 277.0	280.0 ± 329.3	0.4474
Intraoperative blood loss (mL)	470.3 ± 285.4	428.5 ± 313.1	0.7165
Mean infusion rate of glucose during surgery (mg/kg/h)	29.6 ± 17.4	68.0 ± 14.8	< 0.0001*
Mean infusion rate of insulin during surgery (mU/kg/h)	0.2 ± 1.2	31.5 ± 13.5	< 0.0001*

Data are expressed as mean ± SD

*GI* glucose-insulin, *BMI* body mass index, *SBP* systolic blood pressure, *DBP* diastolic blood pressure, *HR* heart rate, *BUN* blood urea nitrogen, *eGFR* estimated glomerular filtration rate

*P < 0.05 compared between the groups

**Fig. 1:**
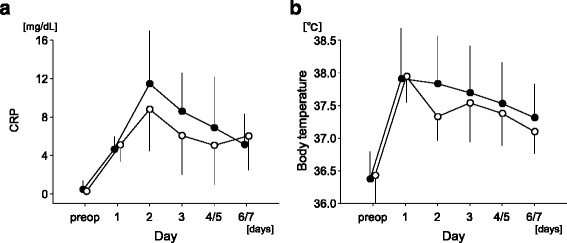
Time course of CRP concentration (**a**) and body temperature (**b**) in the control group (closed circles) and the GI group (open circles). Data are expressed as mean ± SD. Day postoperative day, CRP C-reactive protein, GI glucose-insulin. No significant difference was found between the groups

**Fig. 2:**
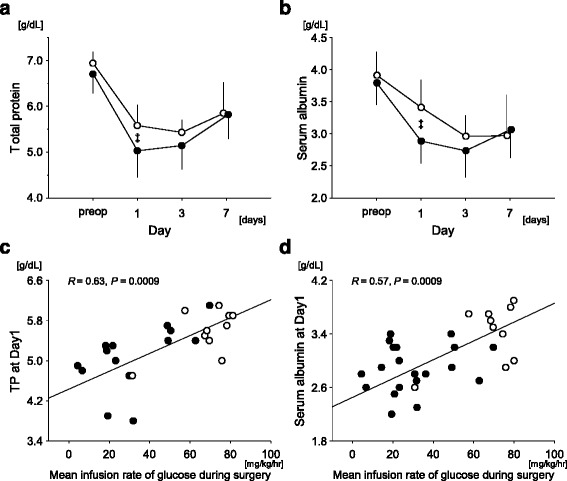
Time course of total protein (**a**) and serum albumin (**b**) concentrations and relationships between the mean infusion rate of glucose during surgery and TP concentrations at Day1 (**c**) and serum albumin concentrations at Day1 (**d**) in the control group (closed circles) and the GI group (open circles). Data are expressed as mean ± SD. Day postoperative day, TP total protein, GI glucose-insulin. ‡*P* < 0.05 compared between the groups

Time course of the CRP concentration and body temperature are shown in Fig. 1. No significant difference was observed between the groups in the CRP concentration and body temperature. Regarding total protein and serum albumin concentrations, significant differences were observed at Day1 between the groups (Fig. 2a, b). Furthermore, significant correlations were observed between the mean infusion rate of glucose during surgery and TP at Day1 (*R* = 0.63, *P* = 0.0009; Fig. 2c) and serum albumin at Day1 (*R* = 0.57, *P* = 0.0009; Fig. 2d), despite no significant relationships found before surgery.

The overall postoperative complications (they overlapped) and hypoalbuminemia in each group are shown in Table 2. Significant difference was observed between the two groups in the incidence of hypoalbuminemia (*P* = 0.0049). However, no significant difference was found in the incidence of hypotension, necrosis of a reconstructed flap, SSI, pneumonia or bacteremia, number of patients who had postoperative complications, and the days required until discharge.

**Table 2:** Postoperative hypoalbuminemia and complications in each group

	Control group (*N* = 20)		GI group (*N* = 10)		*P* value
Postoperative hypoalbuminemia	17		4		0.0122*
Postoperative complications (overlapping)	Hypotension	6	Hypotension	1	0.1976
	Necrosis of a flap	5	Necrosis of a flap	1	0.3104
	SSI (including MRSA)	5	SSI (including MRSA)	1	0.3104
	Pneumonia	1	Pneumonia	2	0.2122
	Bacteremia	1	Bacteremia	0	0.3628
Number of patients who had postoperative complications	13		4		0.1928
Days until discharge	85.6 ± 44.4		68.8 ± 22.5		0.2712

*GI* glucose-insulin, *SSI* surgical site infection, *MRSA* methicillin-resistant *Staphylococcus aureus*

*P < 0.05 between the groups

Results of the multiple linear regression analysis are shown in Table 3. Mean infusion rate of glucose during surgery was independently associated with reduction of both TP and albumin concentrations from the control to Day1. Intraoperative blood transfusion was also associated with reduction of albumin.

**Table 3:** Multiple linear regression analysis

		Reduction of TP from the control to Day1	Reduction of albumin from the control to Day1
Age	*β*	0.0617	− 0.0001
	*P*	0.7899	0.9995
BMI (kg/m^2^)	*β*	− 0.3238	− 0.0474
	*P*	0.1020	0.7838
Operation time (min)	*β*	0.2020	0.0190
	*P*	0.4621	0.9411
Total fluid volume (mL)	*β*	0.4434	0.4326
	*P*	0.2779	0.2886
Intraoperative blood loss (g)	*β*	0.4543	0.1687
	*P*	0.0730	0.5044
Intraoperative blood transfusion (mL)	*β*	− 0.4543	− 0.4872
	*P*	0.0699	0.0307*
Mean infusion rate of glucose during surgery (mg/kg/h)	*β*	− 0.4279	− 0.5047
	*P*	0.0427*	0.0288*
Mean infusion rate of insulin during surgery (mU/kg/h)	*β*	0.0211	0.1764
	*P*	0.9183	0.4462

Italic typescript indicates statistical significance

*TP* total protein, *Day1* postoperative day 1, *BMI* body mass index, *β* standardized partial regression coefficient

## Discussion

In the current study, we expected anti-inflammatory effects of tight glycemic control using GI infusion that have been demonstrated in heart surgery [10] and in critically ill patients [9]. However, the GI infusion did not affect CRP concentrations and body temperature. Instead, it preserved higher TP and albumin concentrations than control subjects. Positive linear relationships were observed between the mean infusion rate of glucose during the surgery and both TP and albumin concentrations of Day1. Furthermore, the mean infusion rate of glucose during the surgery was an independent factor affecting the reduction in both TP and albumin concentration from the control to Day1. Therefore, it is additional glucose, but not insulin, that preserved TP and albumin concentrations.

It rarely induces hypoglycemia during surgery even if only lactate or acetate Ringer’s solution that does not contain glucose is employed. However, it has been demonstrated that both breakdown of protein and fat synthesis are reduced by using glucose-containing solution compared with normal saline solution during abdominal surgery [14], and that supplementation of a small dose of glucose in acetate Ringer’s solution (1% *W*/*V*) during surgery inhibits breakdown of proteins [15]. Therefore, intraoperative energy requirements were compensated by breakdown of proteins and facilitated fat synthesis under the condition of decreased insulin secretion, when intraoperative fluid was solely managed with solutions that did not contain glucose.

It has been reported that continuous infusion of 0.25 μg/kg/h remifentanil inhibited pneumoperitoneum-induced secretion of cortisol and ACTH but not that of catecholamines [16]. In the present study, we should have measured these hormones and compared them between the two groups to secure endocrine and adrenergic responses levels. Thus, we cannot exclude the possibility that remifentanil was not able to inhibit endocrine and/or adrenergic responses in this kind of surgery. In addition, low-dose dopamine, which is widely employed to increase renal blood flow in elderly patients, has been reported to induce insulin resistance [17]. It should be further noted that intraoperative insulin resistance was aggravated by low-dose dopamine.

The present study has limitations. First, we should have measured 3-methylhistidine, which is solely derived from catabolism of skeletal muscle [15], in order to observe direct evidence of protein sparing. Second, we should have measured several inflammatory cytokines, such as IL-6, IL-8, or TNF-α, to clarify an association between GI infusion and inflammatory responses. Third, we should have employed solely lactate Ringer’s solution which contains no glucose in the control group, because even a small dose of glucose administration during surgery can inhibit breakdown of proteins [15]. Instead, we have compared parameters between the two groups and performed multiple linear regression analysis by using mean infusion rate of glucose. Fourth, attention should be paid to evaluation of our results because of the relatively small number of patients enrolled. A larger scale study is required in the future to confirm the current findings.

## Conclusion

Application of additional glucose during major oral and maxillofacial surgery preserved serum albumin concentration; however, it did not lead to less postoperative complications and less days until discharge.

## Abbreviations

ACTH: Adrenocorticotropic hormone
BMI: Body mass index
BP: Blood pressure
CRP: C-reactive protein
Day: Postoperative day
GI: Glucose-insulin
IL: Interleukin
MRSA: Methicillin-resistant *Staphylococcus aureus*
NADPH: Nicotinamide adenine dinucleotide phosphate
SSI: Surgical site infection
TNF: Tumor necrosis factor
TP: Total protein
